# Five-year effect of sequential argon-Nd:YAG prophylactic laser peripheral iridotomy on corneal endothelial cell density in Japanese eyes with narrow angles

**DOI:** 10.1007/s10384-025-01211-5

**Published:** 2025-05-26

**Authors:** Koichi Mishima, Naomi Mataki, Hiroshi Murata, Shinichiro Ohtani, Hiroshi Sakai, Atsuo Tomidokoro, Makoto Aihara, Kazunori Miyata, Aiko Iwase, Makoto Araie

**Affiliations:** 1 Department of Ophthalmology, Kanto Central Hospital, 6-25-1 Kamiyoga, Setagaya-ku, Tokyo 158-8531 Japan; 2 Tajimi Iwase Eye Clinic, Gifu, Japan; 3 Department of Ophthalmology, Center Hospital of the National Center for Global Health and Medicine, Tokyo, Japan; 4 Miyata Eye Hospital, Miyazaki, Japan; 5 Urazoe Sakai Eye Clinic, Okinawa, Japan; 6 Tomidokoro Eye Clinic, Tokyo, Japan; 7 Department of Ophthalmology, Graduate School of Medicine, University of Tokyo, Tokyo, Japan; 8 Kanagawa Dental University Yokohama Clinic, Kanagawa, Japan

**Keywords:** Corneal endothelial cell, Sequential argon-Nd:YAG laser peripheral iridotomy, Narrow angle

## Abstract

**Purpose:**

To evaluate the effects of prophylactic sequential argon- Nd:YAG laser peripheral iridotomy (pLPI_sequential_) on the corneal endothelial cell density (CECD) over 5 years in Japanese eyes with narrow angles (NA-eyes).

**Study design:**

Prospective observational study

**Methods:**

The CECD of NA-eyes before and after pLPI_sequential_ and of untreated NA-eyes were assessed annually over 5 years with non-contact specular microscopy. Routine ophthalmic examinations and measurements using anterior-segment imaging devices were performed at baseline. The time courses of the CECD were analyzed using a multivariable linear mixed-effect model and factors obtained at baseline.

**Results:**

Sixty-nine pLPI_sequential_-treated NA-eyes (69 subjects; mean age, 68.9 years) and 67 pLPI_sequential_-untreated NA-eyes (67 subjects; mean age, 64.4 years) were enrolled. In the pLPI_sequential_-untreated NA-eyes, no baseline factors were correlated significantly with the time course of the CECD, and its decline rate − 4.7 (95% Confidence interval (I: − 13.3 to 4.0) cells/mm^2^ was not significant (p = 0.267). In the pLPI_sequential_-treated NA-eyes, the CECD declined with marginal significance − 12.2 (− 24.7 to 0.3) cells/mm^2^/year (p = 0.0513) over 5 years. Higher laser energy used, thicker iris, and shallower central anterior chamber depth (cACD) at baseline were significantly negatively correlated with the post-laser CECD (p = 0.0092, 0.0119, and 0.0158). No significant difference was seen in the baseline factors-adjusted CECD decline rate (p = 0.262) between both the groups.

**Conclusion:**

Prophylactic sequential argon-Nd:YAG pLPI_sequential_ had no clinically significant effects on the time course of the CECD over 5 years in Japanese NA eyes. However, higher laser energy used, thicker iris, and shallower cACD significantly negatively affected the post-laser CECD.

## Introduction

Primary angle-closure glaucoma (PACG) has the highest prevalence in, and is a major cause of blindness and low vision in East Asia [1–5]. Laser peripheral iridotomy (LPI) has been the conventional first-line therapy for patients with suspected primary angle closure (PACS) and those with primary angle closure (PAC) and PACG [6]. Previous studies show that LPI can widen the anterior chamber (AC) angle, reduce the intraocular pressure (IOP) in PACS eyes, and partly reduce the risk of developing PAC or PACG and future acute angle closure in PACS [7–10]. Although LPI is a relatively safe procedure, there is a potential long-term risk of corneal decompensation [11–14]. Wang et al. report that about two-thirds of cases of corneal decompensation after argon LPI were from Japan [11], and LPI using argon laser alone (argon-LPI) is a major cause of bullous keratopathy in Japan [12–14], suggesting that the corneal endothelium in Japanese eyes may be more vulnerable to LPI-induced insults. However, pulsed neodymium-doped yttrium aluminum garnet (Nd:YAG) laser with high-power density requires fewer spots of shorter duration and less energy for iris penetration [15]. The safety of Nd:YAG prophylactic LPI application to the corneal endothelium is reported [11], confirmed in a recent 6-year prospective study [16]. However, Nd:YAG laser alone may not work well in eyes with dark irides and sequential argon-Nd:YAG LPI [17] or sequential continuous wave Nd:YAG LPI [18] has been used widely in eyes with dark irides including Japanese eyes [17–20]. In a 3-year prospective study, Kumar et al. report a 3.8% decrease in the CECD 3 years after prophylactic sequential argon-Nd:YAG LPI (pLPI_sequential_) in Singaporeans, which did not differ significantly from the untreated fellow eyes [21]; one retrospective study reports a 2.8% CECD reduction 7 years after laser in Japanese patients [22]. However, Lim et al. report two cases of corneal decompensation 3.3 and 11.2 years after pLPI_sequential_ in Singaporeans [23].

Since there is still a relative paucity of information on the long-term safety of pLPI_sequential,_ which uses higher total laser energy than Nd:YAG LPI, it seems important to establish its effects on the CECD, especially in Japanese eyes in which argon LPI is reported to be a major cause of bullous keratopathy [12–14]. We conducted a 5-year prospective observational study of the time course of CECD and related factors in pLPI_sequential_-treated eyes with narrow drainage angles and those followed without pLPI_sequential_ treatment.

## Subjects and methods

New patients who were seen between March 2008 and February 2011 at the outpatient clinic of the Department of Ophthalmology, University of Tokyo Hospital; the Tajimi Iwase Eye Clinic, Gifu, Japan; the Miyata Eye Hospital, Miyazaki, Japan; and the Department of Ophthalmology, University of the Ryukyus Hospital, Okinawa, Japan and who satisfied the inclusion criteria in at least one eye and were thought to be eligible for a 5-year observational follow-up study were recruited. The study protocol followed the tenets of the Declaration of Helsinki and the institutional ethics boards of each institute approved the study (University of Tokyo No1841, Tajimi Iwase Eye Clinic tashibyouNo294, Miyata Eye Hospital CS2007-026, University of Ryukyus H20-5-1). All patients provided written informed consent before enrolling, after the study protocol was explained fully and the patients’ understanding was confirmed. 

The inclusion criteria were (1) peripheral AC depth (ACD) that equaled a quarter of the peripheral corneal thickness or shallower according to the van Herick method temporally and the questioned eye was thought to be possibly occludable on gonioscopy, erformed according to the following procedure described below; (2) a best-corrected visual acuity (BCVA) over 20/25. The exclusion criteria were (1) history of ocular surgery including laser procedure; (2) history of acute angle closure OU and corneal diseases or iritis in the candidate eye; (3) glaucomatous optic neuropathy and/or corresponding glaucomatous visual field damage OU; (4) IOP over 21 mmHg OU; (5) topical ocular hypotensive therapy in the candidate eye; (6) observable presence of corneal disorders such as conra guttae and/or exfoliation material on the pupillary margin and lens surface by slit-lamp microscopy and/or specular microscopy OU; (7) clinically relevant senile cataract in the study candidate eye, and (8) presence of self-reported diabetes mellitus and/or treated systemic hypertension. At enrollment, the study subjects underwent measurements of the BCVA and refraction, biomicroscopic examination, IOP measurements using Goldmann applanation tonometry, and static and dynamic gonioscopy OU. The central ACD and axial length were measured using the IOLMaster (Carl Zeiss Meditec) and the central corneal thickness (CCT) and corneal endothelial cell density (CECD) using specular microscopy. Anterior-segment imaging was performed using anterior-segment-optical coherent tomography (AS-OCT: Visante OCT, Carl Zeiss Meditec) and ultrasound biomicroscopy (UBM). Experienced ophthalmologists performed gonioscopy using a narrow slit-lamp beam to prevent light from entering the pupil under dim light. First, the visibility of the trabecular meshwork was checked in the primary eye position in the four (superior, temporal, inferior and nasal) quadrants and then the peripheral AC angles were graded according to Shaffer’s grading system using a Goldmann two-mirror gonioscope (Haag-Streit Inc.) and avoiding inadvertent compression. The presence of peripheral anterior synechiae (PAS) exceeding the scleral spur was further confirmed using a compression technique whenever necessary. The visual field was measured using the Humphrey Field Analyzer Humphrey Field Analyzer 24-2 Swedish Interactive Thresholding Algorithm-Standard program (HFA 24-2, Carl Zeiss Meditec).

### Specular microscopy

The CECD (cells/mm^2^) and CCT were measured using an autoanalysis of non-contact specular microscope (SP-2000P, Topcon). The measurements were repeated 3 times and the average was adopted. The central ACD (cACD) values used for the current analysis was the difference between the CCT and central ACD values obtained using the IOLMaster.

### Examinations using anterior-segment imaging devices

An experienced ophthalmologist performed AS-OCT using the Visante^®^ OCT (Carl Zeiss Meditec) in a dark room (<3 lux) after a 3-minute acclimation period and repeated the imaging 3 minutes after the room was lightened to 1,000 lux. A horizontal B-scan image was obtained. During the examination, patients were encouraged to open their eyes as wide as possible and, if necessary, the examiner gently helped them keep their eyes open. Inadvertent compression of the eye was carefully avoided. B-scan images were analyzed using software built into the OCT instrument, and the lens vault (LV) and AC width were measured.

An experienced ophthalmologist obtained cross-sectional UBM images of the peripheral AC using the UD-1000 (Tomey with a 40-MHz transducer probe at the 12 (superior), 3 (nasal or temporal), 6 (inferior), and 9 (temporal or nasal) o’clock positions of the eye with the patient in the supine position and following the standard procedure [24, 25]. UBM assessments were carried out in a dark room (<3 lux) after a 3-minute acclimation period and repeated in the same manner 3 minutes after the room was lightened to 1000 lux. A 9 x 6-mm field was imaged and transferred to a personal computer for analysis. The images transferred to a personal computer were analyzed using originally developed software [24, 25]. An experienced ophthalmologist masked to other information relating to the images of all UBM examinations determined the presence of irido-trabecular contact (ITC) and plateau iris configuration. The UBM parameters used in the current study were the angle-opening distances at 500 µm (AOD500), angle recess area, trabecular-iris angle, iris-zonule distance, angle between the posterior corneal surface and the anterior surface of the ciliary body, iris thickness at 1,000 µm (IT1000) and iris convexity (Table 1) [24–26].

**Table 1:** Definition of ultrasound biomicroscopic parameters

Parameters	Definition
Angle-opening distances at 500 µm	Distances between the posterior corneal surface and the anterior iris surface measured on a line perpendicular to the trabecular meshwork from point 500 µm anterior to the scleral spur (SS)
Angle recess area	Triangular area bordered by the anterior iris surface, corneal endothelium, and line perpendicular to the corneal endothelium drawn from point 750 µm anterior to the SS to the iris surface
Trabecular-iris angle	The angle measured with the apex in the iris recess and the arms of the angle passing through a point on the trabecular meshwork 500 µm anterior to the SS
Iris-zonule distance	The posterior chamber depth measured from the posterior iris surface to the first visible zonular fiber at the point just clearing the ciliary process
Angle between the posterior corneal surface and the anterior surface of the ciliary body (TCA)	A circle with a 1000-, 2000-, 3000-µm radius centered on the SS was drawn and the following points of intersection were determined: the posterior corneal surface, the anterior and posterior surface of the iris, and the anterior surface of the ciliary body. TCA was measured on the circle with a 1000-µm radius
Iris thickness at 1000 µm (IT1000)	IT1000 was measured between the anterior and posterior surface at the point on the circle with 1,000 µm.
Iris convexity (IC)	The IC was measured as the distance between the point of greatest convexity on the posterior iris surface and the line going through the most peripheral point and most central point of the posterior iris surface.

### Follow-up schedule

The interval between ophthalmologic examinations depended on the condition of the study and fellow eyes and ranged between 3 and 6 months; specular microscopy and gonioscopy were done at 1-year intervals for 5 years.

Indication for pLPI_sequential_ and procedure.

PACS was defined as an eye with three or more quadrants of gonioscopic angle ITC defined as the inability to visualize the pigmented trabecular meshwork based on static gonioscopy in the primary eye position, IOP below 21 mmHg, absence of PAS and glaucomatous optic neuropathy and or visual field defects, and PAC in an eye meeting the criteria for PACS but with PAS and/or IOP of 22 mmHg or higher. The indication for pLPI_sequential_ was mainly the presence of PACS or PAC; however, eyes in which a portion of the eye had gonioscopic ITC in two quadrants or fewer, or eyes with ITC at 2 or more quadrants observed on UBM under dark conditions also were included in the indication according to the discretion of each attending glaucoma subspecialist, who considered patients’ systemic or other ocular and/or gonioscopic findings, patients’ accessibility to an eye care facility, and/or their levels of understanding of the disease. Whenever pLPI_sequential_ was planned, AS-OCT and UBM examinations were scheduled within 1 month of the pLPI_sequential_. After written consent for pLPI_sequential_ was obtained, one drop of 2% pilocarpine and 1% apraclonidine were instilled at an interval of 5 minutes. Thirty minutes later, pLPI_sequential_ was performed in the peripheral three-fourths of the iris between the 10 and 2 o’clock positions wherever the iris appeared relatively thinner using a standard protocol and one drop of 1% apraclonidine was instilled thereafter. These subjects were followed as the pLPI_sequential_-treated group. Among subjects who met the inclusion criteria and agreed to participate in the 5-year prospective observational study, those who were not indicated for a pLPI_sequential_ procedure, or those who preferred not to undergo pLPI_sequential_ were followed as the pLPI_sequential_-untreated group.

### Statistical analysis

Tagawa et al. studied measurement repeatability of a Topcon specular microscope (SP-1000) and report that standard deviation of measured CECD, coefficient of variation (CV) and % of hexagonal cells (%HEX) was at most 9, 18, and 28 % of the mean values obtained [27], indicating that measurement reproducibility was better for CECD than CV or HEX, and more suited for following the same eyes as far as autoanalysis results of Topcon specular microscope areas concerned. The results obtained for CECD indicate that 50 or more cases completed the 5-year follow-up would be sufficient to detect the reported CECD decline rate in normal eyes, i.e., 2.5%/5 years or slightly more [16, 21, 28–30] with an α error of < 0.05 and β = 0.90.

Chronologic changes in the CECD in both groups were analyzed using the Wilcoxon signed-rank test. Factors associated with changes in the CECD were analyzed using a multivariable linear mixed-effect model with the time changes of the CECD as target variables. Explanatory variables were the time that elapsed after pLPI_sequential_, age, and ocular parameters which were possibly related with pLPI_sequential_-caused damage on the corneal endothelium such as, cACD, CCT, AOD500, IT1000, and LV under light conditions; presence of PAC or PACS; number of quadrants with UBM-determined ITC/eye [31]; those with UBM-determined plateau iris configuration [32] in eyes under light conditions; total laser energy used; and interaction between the time elapsed and other explanatory variables.

Although a longer time course of the CECD change is not suited for a linear model analysis [33], the time course of CECD changes during the 5-year period after pLPI_sequential_ was shown in Table 3 of the result section looked linear rather than exponential, and a linear model was considered more suited for analyzing the current results as in the previous study [21]. The optimal linear model was selected among all possible combinations of predictors (2^*(*number of explanatory variables)^) based on the second –order, bias-corrected Akaike Information Criterion index. The use of a model selection method is recommended to improve the model fit by removing redundant variables [34]. Statistical analyses were performed using the *R* software (version 3.6.3; http://www.r-project.org/). P < 0.050 was considered as significant.

## Results

Of the 267 subjects screened between March 2008 and February 2011, a total of 136 subjects agreed to be included in a 5-year prospective observational study including UBM examination at baseline and examination using a specular microscope at 1-year intervals for 5 years with written informed consent. Among them, 69 eyes of 69 subjects in the treated and 67 eyes of 67 subjects in the untreated group were included for a prospective observational study of the pLPI_sequential_-(Table 2). During 5-year prospective observation in the pLPI_sequential_-treated group, 8 patients (2 underwent cataract surgery, 2 withdrew consent, 1 due to systemic disease, 1 relocated, and 2 for unknown reasons) left the study at 3 years and seven (2 underwent cataract surgery, 1 due to systemic disease, 1 died, 1 relocated, 1 withdrew consent, and 1 for an unknown reason) at 4 years. In the pLPI_sequential_-untreated group, five patients (1 underwent pLPI_sequential_ and 1 cataract surgery, 1 due to systemic disease, 1 relocated, and 1 for an unknown reason) left the study at 3 years and three (1 underwent LPI, 1 withdrew consent, 1 for an unknown reason) left the study at 4 years. The mean CECDs at the 5-year follow-up are shown in Table 3 and no significant differences in the CECDs from baseline were seen in both groups. When all CECD data during the 5-year follow-up were analyzed using a multivariate linear mixed-effect model, the CECD declined with marginal significance by − 12.2 (95% confidence interval: − 24.7 to 0.3) cells/mm^2^/year (0.45%/year on an average) (p =0.0513) and higher laser energy, thicker iris (IT1000), and shallower cACD (p=0.0448, 0.0121, and 0.0142, respectively) were significantly negatively correlated with the CECD (Table 4). The same analysis was conducted that included only the data obtained between 1 and 5 years after pLPI_sequential_. The CECD showed a tendency to decline {− 11.7 (− 25.3 to 2.0) cells/mm^2^/year, p=0.0874} and higher laser energy, thicker iris, and shallower baseline cACD (p=0.0092, 0.01119, and 0.0158, respectively) were significantly negatively correlated with the CECD (Table 4). In the pLPI_sequential_-untreated group, the CECD decline rate over 5 years was − 4.7 (− 13.3 to 4.0) ells/mm^2^/year (0.18%/year on an average) was not significant (p=0.267), and it did not differ significantly from that of the pLPI_sequential_-treated group after adjustment for differences in the variables included in the analysis (p=0.262), and no explanatory variables were significantly correlated with the CECD (Table 5).

**Table 2:** Baseline demographics of pLPI_sequential_-treated and pLPI_sequential_-untreated eyes

	pLPI_sequential_-treated eyes	pLPI_sequential_-untreated eyes	*P* Value
Subjects/eyes	69/69	67/67	
Male/female	14/55	6/61	0.0001
Age (years)	68.9 ± 8.1	64.4 ± 10.1	0.0065
Refractive error (diopters)	0.88 ± 1.91	1.16 ± 1.42	0.72
IOP (mmHg)	14.5 ± 3.3	14.5 ± 2.5	0.90
cACD (mm)	1.99 ± 0.22	2.03 ± 0.24	0.10
Axial length (mm)	22.5 ± 0.8	22.7 ± 0.8	0.0752
Laser energy used for pLPI_sequential_ (mJ)	749.2 ± 322.6	–	–
PAC or PACS eyes	41/69	30/67	0.0905
AOD500 (mm)	0.12±0.10	0.22±0.07	0.0001
IT1000 (mm)	0.37±0.06	0.39±0.05	0.0481
Quadrants with appositional ITC/eye	1.18±1.09	0.93±1.18	0.0834
Quadrants with plateau iris configuration/eye	0.10±0.39	0.30±0.70	0.0432
Lens vault (μm)	953±218	858±185	0.0213
Anterior chamber width (μm)	11267±355	11374±382	0.0964

The data are expressed as the means ± standard deviations. *P* values are the results of comparison between the treated and untreated groups (Wilcoxon rank sum test or χ^2^ test)

*pLPI*_*sequential*_* treated eyes* eyes treated with prophylactic sequential argon-Nd:YAG laser peripheral iridotomy, *pLPI*_*sequential*_* untreated eyes* eyes not treated with prophylactic sequential argon-Nd:YAG LPI, *IOP* intraocular pressure, *cACD* central anterior chamber depth measured using the IOLMaster (Carl Zeiss Meditec, Dublin, CA) minus central corneal thickness, *PACS* eyes with three or more quadrants of gonioscopic irido-trabecular contact defined as the inability to visualize the trabecular meshwork based on static gonioscopy in the primary eye position, *IOP* below 21mmHg, absence of peripheral anterior synechiae (PAS) and glaucomatous optic neuropathy, *PAC* eyes meeting the criteria for PACS but with PAS and/or IOP of 22mmHg or higher, *AOD500* angle-opening distances at 500 µm, *IT1000* iris thickness of 1000 μm from the scleral spur under light conditions, *appositional ITC* appositional irido-trabecular contact determined based on the ultrasound biomicroscopic images, plateau iris configuration, determined according to the definition of Kumar et al. [32]; lens vault, determined with anterior-segment optical coherence tomography (Visante® OCT, Carl Zeiss Meditec, Dublin, CA)

**Table 3:** Corneal endothelial cell density of pLPI_sequential_-treated and pLPI_sequential_-untreated eyes

	pLPI_sequential_-treated eyes		pLPI_sequential_-untreated eyes	
	CECD (cells/mm^2^)	*p* value	CECD (cells/mm^2^)	*p* value
Baseline	2688±333		2574±418	
1 year	2663±344 (− 0.65±8.37)*	0.37	2625±360 (2.25±9.16)*	0.25
2 years	2643±308 (− 1.46±8.92)	0.455	2615±391 (2.80±8.56)	0.12
3 years	2657±305 (− 0.73±7.73)	0.94	2594±406 (1.69±9.58)	0.27
4 years	2620±313 (− 1.88±9.20)	0.11	2563±404 (0.85±12.22)	0.98
5 years	2597±305 (− 1.36±8.30)	0.22	2608±429 (1.78±10.62)	0.26

The data are expressed as the means ± standard deviations (percent change from the baseline)

*CECD* corneal endothelial cell density, *pLPI*_*sequential*_ treated eyes, eyes treated with prophylactic sequential argon-Nd:YAG laser peripheral iridotomy, *pLPI*_*sequential*_* untreated eyes* eyes without prophylactic sequential argon-Nd:YAG LPI

*P* values indicate statistical differences between entry and previous year CECD (Wilcoxon signed-rank test). No significant differences in the CECD at each time point was seen between the pLPI_sequential_-treated and pLPI_sequential_-untreated eyes (*p*>0.30)

^*^Intergroup difference is p=0.0105 using the Wilcoxon rank-sum test, but no significance was seen after Bonferroni’s correction for multiple comparisons

**Table 4:** Mixed-effect model estimates of risk factors for 5 year changes in the CECD* in pLPI_sequential_-treated eyes

	Estimate	SE	*p* value
Intercept	2399 2408*	345 352*	<0.0001 <0.0001*
cACD at baseline	433.9 435.0*	171.2 174.3*	0.0142 0.0158
IT1000 (light) at baseline	− 1682 − 1718*	645 659*	0.0121 0.0119*
Laser energy used for pLPI_sequential_	− 0.238 − 0.356*	0.116 0.132*	0.0448 0.0092*
Time (years after pLPI_sequential_)	− 12.2 − 11.7*	6.2 6.8*	0.0513 0.0874*

*CECD* corneal endothelial cell density (/mm^2^), *pLPI*_*sequential*_ prophylactic sequential argon-Nd:YAG laser peripheral iridotomy, *pLPI*_*sequential*_ energy (mJ) total laser energy used, *cACD* (mm) central anterior chamber depth measured using the IOLMaster (Carl Zeiss Meditec, Dublin, CA) minus the central corneal thickness, *IT1000* (*light*) (*mm*) iris thickness 1000 μm from scleral spur under light conditions

^*^Results obtained using the time changes of the CECD 1, 2, 3, 4, and 5 years after pLPI_sequential_, excluding the CECD measurements before pLPI_sequential,_ as the baseline

**Table 5:** Mixed-effect model estimates of risk factors for 5-year changes in the CECD in pLPI_sequential_-untreated eyes

	Estimate	Standard error	*p* value
Intercept	2270	247	<0.001
cACD at baseline	232.9	123.5	0.0616
AOD500 (light) at baseline	− 559.1	335.4	0.0980
Time (years after baseline)	− 4.7	4.3	0.267

*CECD* corneal endothelial cell density (/mm^2^), *pLPI*_*sequential*_ prophylactic sequential argon-Nd:YAG laser peripheral iridotomy, *cACD* (mm) central anterior chamber depth measured using the IOLMaster (Carl Zeiss Meditec, Dublin, CA) minus the central corneal thickness, *AOD500* (*light*) (*mm*) angle opening distance at 500 μm from the scleral spur under light conditions

## Discussion

The main rationales for the current prospective, observational study of pLPI_sequential_ in Japanese eyes with NAs were the possibility that Japanese eyes might be more vulnerable to argon-LPI-caused insults on the corneal endothelium [11–14], as reported by one previous study. That report followed a time course of CECD prospectively for 3 years after prophylactic sequential argon-Nd:YAG LPI (pLPI_sequential_), and reports that the prevalence of primary angle-closure disease in the Okinawa region of Japan is among the world’s highest [35].

The CECD showed no significant difference between before and 5 years after pLPI_sequential_ treatment, but it slightly declined after pLPI_sequential_ treatment during the 5-year period with marginal significance at a rate of -12.2 cells/mm^2^ (-0.45%)/year, while in eyes with NDAs followed without pLPI_sequential_ treatment, the CECD showed no significant difference over 5 years and its calculated change rate showed no significant difference from that of the pLPI_sequential_ group. In the pLPI_sequential_-untreated group, 59 of 67 subjects completed the 5-year follow-up, suggesting that reported aging-associated CECD change rates of 0.5/year (2.5%/5 years or more) in normal eyes [16, 21, 28–30] could have been well-detected in this group as described in the section of statistical analysis. It seems reasonable that aging-associated CECD decreases in the current pLPI_sequential_-untreated group was smaller than 2.5%/year and might have been detected, if the follow-up period was longer than 5 years. A previous prospective study reports a small decrease in the CECD 3 years after pLPI_sequential_, which did not differ significantly from that in the untreated fellow eyes [21], another retrospective study in Japanese eyes reports a 0.4%/year decrease in the CECD during 7 years after pLPI_sequential_ treatment. Both studies conclude that the CECD change rate observed after pLPI_sequential_ treatment was within the range of aging-associated CECD change rates in normal eyes, which agreed with the current result.

In both datasets of the pLPI_sequential_-treated group, i.e., the one including the baseline (before pLPI_sequential_ treatment) and post-laser data and the other only including post-laser data, higher laser energy, thicker iris (IT1000), and shallower cACD significantly negatively affected the CECD, which suggests that these effects were attributable to the pLPI_sequential_ procedure itself, and that this procedure was not completely harmless to the corneal endothelium. The negative impact of the higher total laser energy and shallower cACD may be explained most reasonably by the direct insults caused by the pLPI_sequential_ procedure itself. The significant negative impact of the thicker iris (IT1000) on the CECD may need investigation. Although a weak positive correlation (Spearman’s correlation coefficient = 0.268, p = 0.040) was seen between the IT1000 and total laser energy used, it is difficult to explain the negative impact of the IT1000 based on the higher laser energy used. If so, the effect of the higher laser energy used should have been represented by the term of laser energy, but not by the term of IT1000 in the multivariate linear mixed-effect model. One possible theory may be that the thicker iris was correlated positively with the pLPI_sequential_-caused inflammatory reactions and subsequent negative impact on the CECD [36]. Another possible mechanism characteristic of pLPI_sequential_ is that the effect of shear stress exerted on the corneal endothelium by altered aqueous flow through a LPI window [37, 38] and the significant negative effect of shallower ACD is compatible with the experimental results [38]. Many previous studies including some recent ones [39–41] report CECD decline after various insults to the eye. Although discussing mechanisms of corneal endothelial damage caused by insults other than pLPI_sequential_ is beyond the scope of this study, inflammation may be one of the common mechanisms, but this hypothesis needs to be confirmed. The small but significant negative impacts of the higher total laser energy, shallower ACD, and thicker iris on the post-pLPI_sequential_ CECD confirms that saving the total laser energy and selecting thinner parts of the peripheral iris are important to decrease the possibility of pLPI_sequential_ caused insults to the CECD. On the other hand, the effect of the presence of appositional ITC on the CECD changes [16, 31], post pLPI_sequential_ treatment, subject’s age reported after phacoemulcification [39] or long-term hard contact lens use [40] are considered relatively minor compared to when the total laser energy used, the cACD, or the iris thickness are accounted for as far as the current subjects are concerned.

The limitations of the current study are that the CECD was measured only in the central cornea, whereas LPI was performed in the peripheral iris. Further assessment of the peripheral cornea near the pLPI_sequential_ site should provide more detailed information on the CECD after pLPI_sequential_ treatment. The pLPI_sequential_-untreated group was not a control group assigned randomly to either treatment or no treatment, but was included to estimate the physiologic aging-related reduction rate of the CECD in Japanese eyes with NAs with a mean age of about 65 years. Thus, sensitivity of an intergroup comparison of an estimate of the time change rate of the CECD after adjustment for the effects of explanatory variables would have been lower than if the treated and untreated groups had been assigned randomly. In the pLPI_sequential_-untreated group, the physiologic aging-related reduction rate of CECD was thought to be smaller than the reported values in normal subjects of about 0.5%/year [16, 21, 28–30], but the exact value itself could not be determined, probably because the sample size was relatively small. Lastly, CV and %HEX could not be included in the current analysis, since both parameters yielded lower reproducibility than CECD and thus, were thought to be not suited for a long-term follow up of the results obtained from the same eye; %HEX was not available in the autoanalysis of the Topcon specular microscope currently used. It is possible that some important risk factors could not be detected [41] and future studies adopting not only CECD, but also CV or %HEX for longitudinal follow may yield more insights into the mechanism of pLPI_sequential_-caused corneal endothelial damage.

In conclusion, although prophylactic sequential argon Nd:YAG laser peripheral iridotomy had potential negative influence on CECD in eyes with higher laser energy used, thicker iris, and shallower cACD, it seemed to have no clinically significant effects on the time course of the CECD over 5 years in Japanese eyes with NAs.
